# Evaluating Antibiotic Resistance in Pediatric UTIs: Five-Year Data from a Tertiary Hospital in Turkey

**DOI:** 10.3390/medicina61030402

**Published:** 2025-02-26

**Authors:** Fedli Emre Kılıç, Osman Küçükkelepçe

**Affiliations:** 1 Training and Research Hospital, Department of Pediatrics, Faculty of Medicine, Adıyaman University, Adıyaman 02040, Turkey; doctoremre2002@gmail.com; 2 Adiyaman Provincial Health Directorate, Adıyaman 02100, Turkey

**Keywords:** empirical treatment, bacterial pathogens, antibiotic resistance, urinary tract infection

## Abstract

*Background and Objectives*: Urinary tract infections (UTIs) are common in children and account for 5–6% of febrile illnesses. This study aims to evaluate the bacterial pathogens isolated from pediatric UTI cases and their antibiotic resistance patterns to inform updated treatment guidelines. *Materials and Methods*: This retrospective study included 2753 children with positive urine cultures treated at Adıyaman Training and Research Hospital from January 2020 to June 2024. Data on patient demographics, bacterial culture results, and antibiotic resistance patterns were analyzed. *Results*: Among the 2753 patients, 71.1% were female and 28.9% were male, with a mean age of 54.6 ± 48.6 months. *Escherichia coli* was the predominant pathogen, isolated in 61.2% of cases, followed by *Klebsiella pneumoniae* (13.3%), *Proteus mirabilis* (9.1%), and *Enterococcus faecalis* (3.5%). Gender-specific differences showed that *E. coli* was more frequently isolated in females (71.7%), while *Proteus* was more common in males (18.5%). Antibiotic resistance analysis revealed high resistance rates to ampicillin (67.4% in *E. coli* and 100% in *Klebsiella*), TMP-SMX (33.2% in *E. coli* and 30% in *Klebsiella*), and cefixime (45.3% in *E. coli*). Amikacin showed the lowest resistance across all pathogens, with only 0.9% resistance in *E. coli*. The resistance to third-generation cephalosporins, particularly ceftriaxone and cefixime, has significantly increased over time, especially in the *Klebsiella* species. *Conclusions*: The results indicate high resistance to ampicillin and TMP-SMX. However, *E. coli* and other pathogens remain susceptible to nitrofurantoin, amikacin, and carbapenems, making these antibiotics viable for empirical therapy. Regional resistance should be considered when selecting treatments for pediatric UTIs to improve outcomes and reduce resistance development.

## 1. Introduction

Urinary tract infections (UTIs) are the second most common cause of infections, accounting for 25% of all infectious diseases. Clinical symptoms such as costovertebral angle tenderness, fever, suprapubic tenderness, frequent urination, burning sensation, urgency, urinary incontinence, and bacteria and inflammatory cells in the urine define a urinary tract infection [1]. UTIs are a bacterial infection that leads to significant mortality, morbidity, and healthcare costs worldwide across all age groups [2]. The main bacterial etiologies of the infection include *Escherichia coli* (*E. coli*), *Klebsiella* species, *Proteus* species, *Pseudomonas aeruginosa*, *Acinetobacter* species, *Enterobacter* species, *Citrobacter* species, *Staphylococcus saprophyticus*, *Enterococcus* species, and *Staphylococcus epidermidis* [3]. It is reported that a single bacterial agent is responsible for 95% of urinary tract infections, with Gram (-) aerobic bacilli from the Enterobacteriaceae family being the most frequent cause. *E. coli* remains the most common pathogen causing symptomatic UTIs and asymptomatic bacteriuria in all age groups, including newborns [4,5].

The prevalence of UTIs during childhood is 3–5% in girls and 1% in boys [6]. UTIs are commonly seen during childhood, especially in infancy and late adolescence [7]. In boys under the age of one, the incidence is five times higher, while it becomes ten times more frequent in girls after that [8]. Diagnosing UTIs in children under one year of age is difficult as they often present with nonspecific symptoms. Furthermore, UTIs in children can present with a wide clinical spectrum, ranging from uncomplicated cystitis to pyelonephritis and sepsis. Despite advances in diagnosis and treatment, UTIs are prone to recurrence [9]. Antibiograms are utilized in managing patients suspected of having UTIs. However, as it takes time to obtain antibiogram results, empirical antibiotic therapy is initiated in patients: the causative agent and the selected antibiotic impact treatment success. Antibiotic resistance is critical to choosing the right drug for empirical antibiotic therapy. It is important to periodically monitor the hospital’s and region’s sensitivity patterns when selecting empirical antibiotics [10]. Nowadays, the first-line antibiotics physicians prefer for patients suspected of having UTIs that include ampicillin–sulbactam, trimethoprim–sulfamethoxazole (TMP-SMX), amoxi-cillin, and oral cephalosporins. Selecting an appropriate empirical antibiotic will significantly reduce morbidity and mortality. Delayed diagnosis and inappropriate and inadequate treatment may lead to long-term kidney damage and hypertension [9]. Understanding antibiotic resistance patterns helps guide effective empirical antibiotic selection and reduces treatment failures. Incorrectly selected empirical therapy not only increases morbidity but also leads to prolonged treatment, repeated outpatient or emergency visits, and increased hospitalization costs [11].

Our literature review found no studies have been conducted on antibiotic resistance in urinary tract infections after 2020 [12]. In this study, we investigated the most frequently isolated microorganisms in childhood UTIs in our region and their antibiotic resistance rates. Our goal is to present the resistance status of microorganisms isolated from urine due to the widespread use of antibiotics, contributing to the current literature. In light of these findings, we aim to highlight the potential need to reevaluate the antibiotics recommended for first-line treatment.

## 2. Materials and Methods

This retrospective study was conducted in pediatric outpatient clinics at Adıyaman University Training and Research Hospital in Turkey, involving children under 18. A total of 2753 children under 18 years old with positive urine cultures were included in the study. All cases were identified between January 2020 and June 2024 at the pediatric outpatient clinics of Adıyaman University. Patient records were retrospectively reviewed, and data on age, gender, admission date, culture results, bacterial species, and antibiogram findings were recorded. No sampling method was used as the study included the entire population. Patients were identified using their unique Turkish identity numbers. Only one positive culture from each patient was included. The confidentiality of the patient’s personal information was maintained. Patients hospitalized or with catheters, except those admitted for day procedures, were excluded from the study.

After standard cleaning procedures, urine samples were collected from young patients without urinary control using sterile urine bags or bladder catheterization, and midstream urine samples were collected from patients with urinary control. Semi-quantitative urine samples were inoculated on Eosin Methylene Blue (bioMérieux, Marcy l’Etoile, France) and 5% sheep blood agar using 4 mm diameter loops and incubated at 37 °C for 18–24 h. UTI diagnosis was based on clinical findings and significant bacteriuria in the culture. In this study, significant bacteriuria was defined as the growth of a single bacterial species at a concentration of 10^5^ c.f.u./mL in midstream or sterile urine bag samples or the growth of a single bacterial species at >10^4^ c.f.u./mL in urine samples collected via transurethral bladder catheterization. Growth of less than 10^5^ c.f.u./mL in midstream or sterile urine bag samples was considered insignificant bacteriuria (negative).

The identification of isolates was performed using colony morphology, Gram staining, IMViC tests (indole, methyl red, Voges-Proskauer, and citrate), and the Vitek 2 Compact fully automated identification system (bioMérieux). Antimicrobial susceptibility testing was performed according to the Clinical and Laboratory Standards Institute (CLSI) guidelines. Susceptibility to various antibiotics, such as ampicillin, amikacin, ceftazidime, cefixime, ciprofloxacin, ceftriaxone, aztreonam, benzylpenicillin, cefazolin, cefoxitin, ciprofloxacin, nitrofurantoin, ertapenem, gentamicin, imipenem, levofloxacin, meropenem, tobramycin, TMP/SMX, piperacillin/tazobactam (Pip/Tazo), amoxicillin/clavulanate, ertapenem, cefepime, cefuroxime, and vancomycin, was evaluated using the automated identification system.

### 2.1. Ethical Approval

In the application for ethics committee approval, it was specified that the study would involve a retrospective examination of digital patient records. Consent from the participants or their guardians was not included in the application because the participants were minors. Consequently, the Non-Interventional Research Ethics Committee of Fırat University (Date: 1 August 2024, Decision no: 2024/11-08) granted approval for the study and waived the need for consent forms. Still, institutional permission was obtained from the Adıyaman University Training and Research Hospital Administration, on the condition that identifying patient information was kept confidential. The study was conducted in accordance with the principles of the Declaration of Helsinki.

### 2.2. Statistical Analysis

The analyses were performed using SPSS (Statistical Package for Social Sciences; SPSS Inc., Chicago, IL, USA) version 22. Descriptive data were presented as *n* and % values for categorical variables and as mean ± standard deviation (mean ± SD) values for continuous variables. Pearson’s Chi-square test was applied to compare categorical variables between groups. The normal distribution of continuous variables was assessed using the Kolmogorov–Smirnov test. The Mann–Whitney U test was used to compare two independent groups. A *p*-value of <0.05 was considered statistically significant.

## 3. Results

A total of 132,557 urine cultures were requested from pediatric outpatient clinics, and growth was detected in 2.07% of these cultures. Of the 2753 patients in the study, 795 (28.9%) were male, and 1958 (71.1%) were female, with bacterial growth observed in all patients’ urine cultures. The average age of the patients was 54.6 ± 48.6 months (min: 1, max: 196). The average age of the male patients was 28.5 ± 36.2 months, while for female patients, it was 65.2 ± 49.1 months, and this age difference was statistically significant (*p* < 0.001). In 8.8% of the patients, Gram-positive bacteria were isolated, while 91.2% had Gram-negative bacteria. The most frequently isolated microorganism was *E. coli*, at 61.2%. This was followed by *Klebsiella* spp. at 13.3%, *Proteus mirabilis* at 9.1%, and *Enterococcus faecalis* at 3.5% (Table 1).

**Figure 1:**
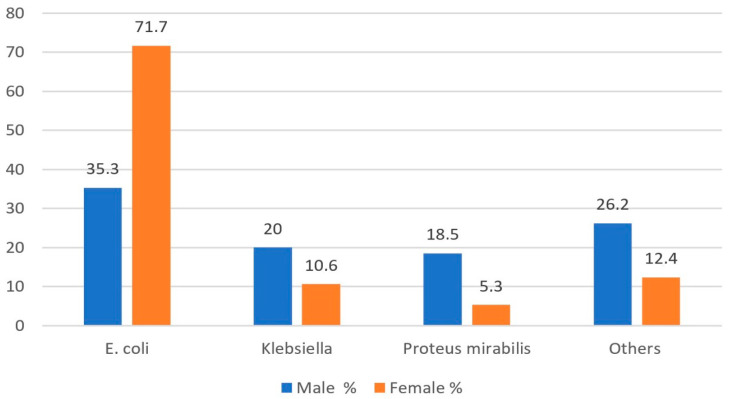
Distribution of bacteria by gender.

When evaluated by gender, *E. coli* was isolated in 35.3% of male patients, *Klebsiella* in 20%, and *Proteus mirabilis* in 18.5%. In female patients, *E. coli* was detected in 71.7%, *Klebsiella* in 10.6%, and *Proteus mirabilis* in 5.3%. There was a statistically significant difference in these distributions (*p* < 0.001) (Figure 1).

**Figure 2:**
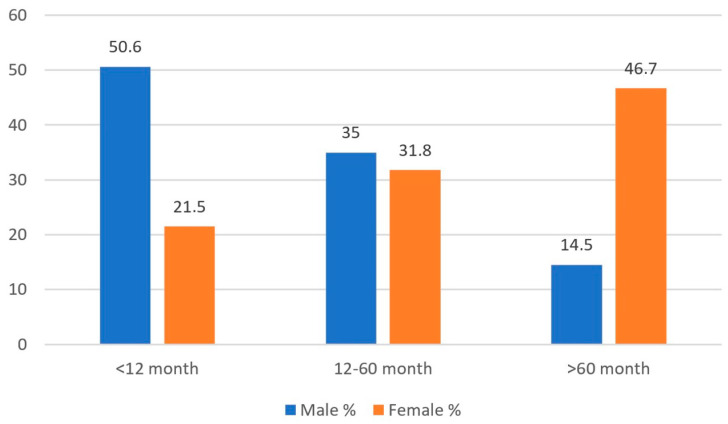
Gender distribution by age in urinary tract infections.

When evaluated by age, 29.9% (*n* = 823) of the patients were under 12 months old, 32.7% (*n* = 1030) were between 12 and 60 months old, and 37.4% were over 60 months old. UTIs were more common in males under 12 months (50.6%) and in females over 60 months (46.7%) (Figure 2).

When analyzed by age, the rate of *E. coli* was 43.1% in children under 12 months, 62.7% in those between 12 and 60 months, and 74.4% in those over 60 months. There was a statistically significant difference between these age groups (*p* < 0.01) (Table 2).

Across all age groups, significant differences were observed in *E. coli*, *Klebsiella*, and *Proteus* rates between male and female patients (*p*1 = 0.001, *p*2 < 0.001, *p*3 < 0.001). *E. coli* was more prevalent in females across all age groups, while *Proteus* was more frequently isolated in males (Table 3).

When examining the resistance patterns of all bacteria isolated in UTIs, the highest resistance rate was found against ampicillin (70.8%). This was followed by resistance to cefazolin, cefixime, and amoxicillin/clavulanate. For the most frequently observed microorganism, *E. coli*, resistance to ampicillin was 67.4%, while resistance to amikacin was 0.9% (Table 4).

## 4. Discussion

Urinary tract infection (UTI) is a common and significant infectious disease in children. UTIs account for 5–6% of infections in children evaluated for fever [13]. Since UTIs are one of the most common diseases, making a quick diagnosis, starting appropriate treatment, and conducting a detailed examination to prevent long-term complications is essential [14]. Therefore, timely initiating empirical treatment with a rapid and accurate diagnosis is crucial when UTIs are suspected. The antimicrobial resistance distribution of microorganisms causing UTIs varies by region and over time [15]. In selecting empirical treatment, the region’s most frequently identified agent and the lowest resistance rate should be considered [16]. Since Gram-negative microorganisms are the most common cause of UTIs in children, the empirical treatment choice should primarily focus on these microorganisms [17].

This study analyzed bacteria isolated from pediatric patients in a tertiary hospital and their resistance to oral and parenteral antibiotics. This aims to contribute to the current guidelines for empirical antibiotic treatment in UTIs. In childhood, including the neonatal period, *E. coli* remains the most common cause of symptomatic UTIs and asymptomatic bacteriuria, accounting for 80% of cases [18]. International studies have also identified *E. coli* as the most common microorganism. For instance, in the study by Vazorous et al., *E. coli* (79.2%) was followed by *Klebsiella* (7.2%) and *Proteus* (5.1%) [19]. At the same time, Baines et al. reported *E. coli* as the most frequent isolate, followed by *Klebsiella* and *Enterobacter* [20]. Similarly, Al Benvan et al. found *E. coli* (67.3%) followed by *Klebsiella pneumoniae* (8.9%), *Proteus* spp. (5.7%), and *Enterococcus* spp. (7.4%) [21]. In studies conducted in Turkey, Kömürlüoğlu et al. identified *E. coli* as the most frequent pathogen (64.1%), followed by *Klebsiella* spp. (17.1%) and *Proteus* spp. (7.4%) [16], while Demir et al. reported *E. coli* (58.9%), *Klebsiella* (17.9%), and *Proteus* (15.8%) [12]. Other studies by Yolbaş et al. [22], Çetin et al. [23], and Çapan et al. [15] *E. coli*, *Klebsiella* spp., and *Proteus* spp. were similarly reported as the most common pathogens. In our study, *E. coli* was the most frequently identified microorganism in urine culture samples (61.2%), followed by *Klebsiella pneumoniae* (13.3%), *Proteus mirabilis* (9.1%), and *Enterococcus faecalis* (3.5%). These findings are consistent with the literature.

The frequency of UTIs in children varies by age and gender. UTI prevalence is highest in male infants during the first three years of life, but this rate significantly decreases as they age. The high rate of UTIs in young male children may be attributed to the presence of the prepuce. In girls, the incidence of UTIs remains similar across all ages, with the risk of infection in females being attributed to the short urethra and its proximity to the anus. Vazouras et al. found that UTIs, with a higher frequency, were most common in girls’ first two years of life [19]. In the study by Demir et al., UTIs were more frequent in males during the first year, while they were more common in females in all other age groups [12]. Konca et al. found that most UTI cases (62.6%) occurred in females, with the highest incidence in male infants during the first three years of life [15]. In the study by Çoban et al., 78.8% of patients with positive urine cultures were female. Although UTIs were more common in males under one year of age, the incidence was higher in females in all other age groups [13]. In our study, UTIs were most common in females (71.1%), with the highest incidence in males during the first 12 months (50.6%) and in females after 60 months of age (46.7%).

Oral treatment for UTIs is as effective as parenteral therapy [24]. Empirical antibiotics commonly selected for oral use include ampicillin, amoxicillin–clavulanate, TMP-SMX, cefixime, cefpodoxime, cefprozil, cefuroxime axetil, and cephalexin. Parenteral therapy options for patients unresponsive to oral treatment include ceftriaxone, cefotaxime, ceftazidime, gentamicin, tobramycin, and piperacillin/tazobactam [25].

Microorganisms that cause urinary tract infections (UTIs) often show high levels of resistance to ampicillin and TMP-SMX, which are frequently chosen in primary healthcare facilities. According to international literature, Shao et al. reported 78.9% resistance to ampicillin and 67% to TMP-SMX in *E. coli* strains [26]. In another study from Taiwan evaluating Gram-negative urine cultures, *E. coli* was found to have a 92% resistance rate to ampicillin [27]. Edlin et al. found ampicillin resistance in 45% of *E. coli* isolates and 24% in TMP/SMX, while resistance in *Klebsiella* was 81% to ampicillin and 15% to TMP-SMX [28]. In the Turkish literature, Konca et al. reported ampicillin resistance rates of 56.4% in *E. coli*, 78.3% in *Klebsiella*, and 42.3% in *Proteus*, while TMP-SMX resistance rates were 43.2% in *E. coli*, 30.4% in *Klebsiella*, and 65.4% in *Proteus* [15]. Kömürlüoğlu et al. found ampicillin resistance in *E. coli* at 68.9% and TMP-SMX resistance at 46.7%, while *Klebsiella* spp. showed 100% resistance to ampicillin and 38.6% to TMP-SMX. *Proteus* spp. exhibited 54% resistance to both ampicillin and TMP-SMX [16]. In the study by Salduz et al., resistance to ampicillin was 71.3% in *E. coli* strains and 80% in *Proteus* strains, with TMP-SMX resistance reported at 52.7% for *E. coli* and 56% for *Proteus* [29]. In our study, resistance to ampicillin was found to be 67.4% in *E. coli*, 100% in *Klebsiella*, and 48.6% in *Proteus*, while TMP-SMX resistance was 33.2% in *E. coli*, 30% in *Klebsiella*, and 44.4% in *Proteus*. Based on these resistance rates, empirical treatment with ampicillin or TMP-SMX would be insufficient without culture results in patients with suspected UTIs. Considering all these studies and the lack of access to culture antibiograms in primary care in Turkey, it is concluded that amoxicillin–clavulanate may be a more effective initial treatment option than ampicillin. While some studies report increasing resistance to amoxicillin–clavulanate over time, this resistance remains significantly lower than ampicillin, as demonstrated in our study.

There is high resistance to second-generation cephalosporins like cefuroxime (CXM), which are commonly used in UTI treatment. A study from Israel found resistance to CXM in 1% of *E. coli* and 22% of *Klebsiella* isolates [30]. Yaşar et al. reported CXM resistance rates of 37% in *E. coli* and 21% in *Klebsiella* [14]. In the study by Tosun et al., resistance to second-generation cephalosporins was 9.8% in *E. coli* strains, 32% in *Klebsiella*, and 41.6% in *Proteus* [31]. Sezgin et al. reported CMX resistance at 21.6% [32]. In the study by Gündüz et al., CXM resistance was 10.2% in *E. coli*, 40% in *Klebsiella*, and 46% in *Proteus* [33]. Similarly, Kömürlüoğlu et al. found resistance to cefuroxime axetil in 34.6% of *E. coli*, 44.8% of *Klebsiella*, and 14.6% of *Proteus* [16]. In our study, resistance to CXM was found to be 44% in *E. coli* and 50% in *Klebsiella*. Given these higher resistance rates compared to other studies, we do not recommend empirical treatment with CXM in suspected UTIs in the Adıyaman region. However, further studies from different regions in Turkey will help clarify the issue and contribute to the broader dissemination of appropriate treatment options.

Resistance to third-generation cephalosporins has also increased. In the study by Konca et al. conducted in the Adıyaman region in 2017, resistance to ceftriaxone was 33.6% in *E. coli*, 38.4% in *Klebsiella*, and 34.7% in *Proteus*, while resistance to cefixime was 36.8% in *E. coli*, 53.8% in *Klebsiella*, and 11.1% in *Proteus* [15]. In the study by Yaşar et al., resistance to ceftriaxone was found 15% in *E. coli*, 17% in *Klebsiella*, and 2% in *Proteus*. In comparison, cefixime resistance was 13% in *E. coli*, 17% in *Klebsiella*, and 7% in *Proteus* [14]. Gürgöze et al. reported resistance to third-generation cephalosporins as 12% in *E. coli*, 21% in *Klebsiella*, and 20% in *Proteus* [34]. Kömürlüoğlu et al. reported cefixime resistance as 31.3% in *E. coli*, 38.2% in *Klebsiella*, and 8.8% in *Proteus* [16]. Cebe et al. found third-generation cephalosporin resistance in *E. coli* to be 12.8% [35]. In the study by Çoban et al., resistance to cefixime was 27% in *E. coli*, 15% in *Klebsiella*, and 6% in *Proteus* [13]. In our study, resistance to parenteral ceftriaxone, a frequently prescribed third-generation cephalosporin, was 38% in *E. coli*, 44.3% in *Klebsiella*, and 13.1% in *Proteus*. Resistance to the oral third-generation cephalosporin cefixime was 45.3% in *E. coli*, 50.8% in *Klebsiella*, and 21.8% in *Proteus*. Compared to the 2017 study by Çapan et al. conducted in the same region, resistance to both ceftriaxone and cefixime generally increased [15]. Based on these results, resistance to third-generation cephalosporins in suspected UTI cases has significantly increased in our region. We recommend that third-generation cephalosporins not be used as first-line treatment, and antibiotics should be chosen based on antibiogram results to prevent unnecessary antibiotic use. It is also suggested that efforts be made to raise awareness among primary and secondary care physicians.

Aminoglycosides are among the most preferred agents for parenteral antibiotic treatment of UTIs. In a study conducted in Iran, resistance to amikacin was found 4% in *E. coli* and 18.2% in *Klebsiella* isolates [36]. In the study by Konca et al., no resistance to amikacin was observed, while resistance to gentamicin was 4.2% in *E. coli*, 13% in *Klebsiella*, and 19.2% in *Proteus* [15]. In the study by Yaşar et al., no resistance to gentamicin or amikacin was found in *E. coli*, *Klebsiella*, or *Proteus* isolates [14]. In the study by Salduz et al., resistance to gentamicin was 6.8% in *E. coli* and 10% in *Proteus*, with no resistance to amikacin reported [29]. Kömürlüoğlu et al. found resistance to amikacin in 16.1% of *E. coli*, 18.9% of *Klebsiella*, and 4.1% of *Proteus* [16]. In our study, resistance to amikacin was 0.9% in *E. coli*, 10.4% in *Klebsiella*, and 3.6% in *Proteus*, while resistance to gentamicin was 8.7% in *E. coli*, 22.7% in *Klebsiella*, and 25.2% in *Proteus*. Based on these results, aminoglycosides are suitable for UTI treatment due to their low resistance rates. However, their ototoxic and nephrotoxic side effects should be considered, and caution should be exercised in their use; overuse may contribute to the development of resistance against these agents.

Fluoroquinolones are commonly used antibiotics in the treatment of UTIs. Tseng et al. reported a ciprofloxacin resistance rate of approximately 7% [27]. A 2013 study from Greece reported that resistance to quinolones increased from 5.7% in 2005 to 8.8% in 2010 [37]. Kömürlüoğlu et al. found ciprofloxacin resistance rates of 23.2% in *E. coli*, 17.2% in *Klebsiella*, and 22.6% in *Proteus* [16]. In a similar study by Konca et al. in Adıyaman in 2016, ciprofloxacin resistance was found to be 6.3% in *E. coli*, 13.6% in *Klebsiella*, and 7.7% in *Proteus* [15]. In our study, resistance to ciprofloxacin was 15.3% in *E. coli*, 23.5% in *Klebsiella*, and 15.2% in *Proteus*. Compared to the 2016 study by Çapan et al., we observed an increase in quinolone resistance, indicating that empirical treatment with ciprofloxacin should be avoided until the antibiogram results are available [15]. We believe this increase is related to the widespread use of fluoroquinolones.

Carbapenems are the most preferred group for parenterally treating UTIs caused by resistant microorganisms. In studies conducted outside of Turkey, Mantadakis et al. found carbapenem susceptibility rates ranging from 98.8% to 100% [38]. In a study conducted in Iran, resistance to meropenem was 1.9% and to imipenem 2.1% in *E. coli* strains, while resistance to meropenem and imipenem in *Klebsiella* was 26.2% and 17.9%, respectively [36]. Yavaşcan et al. reported the highest carbapenem resistance in *E. coli* strains (18.5%) and the lowest in Pseudomonas strains (2.4%) [17]. Mir et al. reported the highest resistance to carbapenems in *Enterobacter* strains (12.5%), while no resistance was observed in *Proteus*, *Klebsiella*, or *Pseudomonas* strains [38]. In the study by Konca et al., meropenem resistance was 0.7%, imipenem resistance was 4.1%, and ertapenem resistance was 4.6% [15]. Kömürlüoğlu et al. reported resistance rates of 1.3% to meropenem, 1.5% to imipenem, and 1.7% to ertapenem in *E. coli* [16]. In our study, resistance to imipenem, meropenem, and ertapenem in the most frequently isolated bacterium, *E. coli*, was found to be 5.6%, 3.8%, and 9.5%, respectively. Although carbapenem resistance was found to be higher than reported in the literature, *E. coli*, the most common cause of UTIs, remains highly susceptible to carbapenems. We recommend considering meropenem for use in multi-resistant hospitalized cases; however, its use should be approached with caution, as overuse may contribute to the development of carbapenem-resistant organisms.

Although nitrofurantoin is active against 96% of *E. coli* isolates, its poor renal parenchymal penetration limits its use in pyelonephritis. However, nitrofurantoin remains an appropriate option for empirical treatment in uncomplicated UTIs, particularly in community settings [39]. In the study by Baines et al., nitrofurantoin resistance was 2% in *E. coli*, 24% in *Klebsiella*, and 98% in *Proteus* strains [20]. Al Benvan et al. reported nitrofurantoin resistance at 4% in *E. coli* strains [21]. In the study by Edlin et al., nitrofurantoin resistance was found to be less than 1% [28]. In the study by Konca et al., nitrofurantoin resistance was found to be 3.3% in *E. coli*, 10% in *Klebsiella*, and 88% in *Proteus* [15]. In the study by Demir et al., nitrofurantoin resistance was found to be 6.4% in *E. coli*, 15.9% in *Klebsiella*, and 64.4% in *Proteus* [12]. In our study, resistance to nitrofurantoin was found to be 1.8% in *E. coli*, 23.1% in *Klebsiella*, and 99.6% in *Proteus*. Similarly to other studies, we found low resistance to nitrofurantoin in *E. coli*. Although our study found a low resistance rate to nitrofurantoin, its potential to adversely affect growth cartilages means that it should be used with caution and considered as a second-line option rather than a first-line empirical therapy [40].

Since this study is retrospective, we were unable to access the patients’ past diagnoses and detailed discharge summaries. Therefore, information regarding urinary tract anomalies, metabolic or neurologic diseases, recurrent versus single UTIs, and previous antibiotic treatment could not be obtained. Due to the cross-sectional design of the study and the fact that it was conducted in only one hospital in Turkey, the generalizability of the results may be limited. Although our study found that resistance to nitrofurantoin, carbapenems, and amikacin was quite low, this may not be sufficient to recommend these agents as first-line empirical therapies. Therefore, it is recommended that further studies be conducted with larger sample sizes and across different regions to more comprehensively assess antibiotic resistance.

## 5. Conclusions

In conclusion, the rapid diagnosis and appropriate treatment of UTIs in children are crucial to preventing long-term complications. As demonstrated in our study, despite the high antibiotic resistance of *E. coli*—the most common cause of UTIs—some antibiotics, including nitrofurantoin, amikacin, and carbapenems, retain significant efficacy. Based on our findings, agents with lower resistance rates—such as amoxicillin–clavulanate—are preferable to ampicillin and TMP-SMX for first-line treatment. Although nitrofurantoin exhibits low resistance, its potential adverse effects on growth cartilage in children warrant its cautious use as a secondary option rather than as a primary empirical therapy. In addition, high bacterial resistance to amoxicillin limits its effectiveness, and although carbapenems-amikacin show low resistance, they should be reserved for multi-resistant cases to minimize the risk of emerging carbapenem and amikacin-resistant organisms. Regional data play a critical role in guiding empirical treatment, and these data should be carefully considered before initiating therapy. Furthermore, a treatment approach based on culture and antibiogram results is a crucial strategy to prevent unnecessary antibiotic use and the development of resistance.

## Figures and Tables

**Table 1:** Isolated microorganisms in pediatric UTI cases.

Microorganism	*n*	%
*Escherichia coli*	1685	61.2
*Klebsiella pneumoniae*	367	13.3
*Proteus mirabilis*	251	9.1
*Enterococcus faecalis*	95	3.5
*Klebsiella oxytoca*	49	1.8
*Enterobacter cloacae*	46	1.7
*Pseudomonas aeruginosa*	43	1.6
*Enterococcus faecium*	38	1.4
*Staphylococcus epidermidis*	25	0.9
*Morganella morganii*	24	0.9
*Staphylococcus haemolyticus*	24	0.9
*Streptococcus pyogenes*	21	0.8
*Staphylococcus aureus*	20	0.7
*Citrobacter*	16	0.6
*Enterobacter aerogenes*	16	0.6
*Streptococcus agalactiae*	13	0.5
*Staphylococcus simulans*	7	0.3
*Acinetobacter*	6	0.2
*Providencia rettgeri*	5	0.2
*Cedecea*	2	0.1

**Table 2:** Distribution of microorganisms by age group.

	<12 Months	12–60 Months	>60 Months
By Gram stain	*n*	%	*n*
	Gram-positive	82	10.0
	Gram-negative	741	90.0
By bacterial species	*E. coli*	355	43.1
	*Klebsiella*	258	31.3
	*Proteus mirabilis*	34	4.1
	Other	176	21.4
	<12 months	12–60 months	>60 months
	*n*	%	*n*
By Gram stain	Gram-positive	82	10.0
	Gram-negative	741	90.0

**Table 3:** Distribution of microorganisms by age and gender.

	Male						Female						
	<12 Months		12–60 Months		>60 Months		<12 Months		12–60 Months		>60 Months		*p*
	*n*	%	*n*	%	*n*	%	*n*	%	*n*	%	*n*	%	
Gram-positive	38	9.5	25	9.0	24	20.9	44	10.5	37	5.9	75	8.2	*p*1 = 0.633 *p*2 = 0.096 ***p*3 < 0.001**
Gram-negative	364	90.5	253	91.0	91	79.1	377	89.5	585	94.1	840	91.8	
*E. coli*	156	38.8	82	29.5	43	37.4	199	47.3	482	77.5	723	79.0	***p*1 = 0.001** ***p*2 < 0.001** ***p*3 < 0.001**
*Klebsiella*	125	31.1	24	8.6	10	8.7	133	31.6	27	4.3	48	5.2	
*Proteus mirabilis*	26	6.5	104	37.4	17	14.8	8	1.9	55	8.8	41	4.5	
Other	95	23.6	68	24.5	45	39.1	81	19.2	58	9.3	103	11.3	

*p*1 = Comparison of <12 months of male vs. female, *p*2 = Comparison of 12–60 months of male vs. female, *p*3 = Comparison of >60 months of male vs. female.

**Table 4:** Antibiotic resistance rates in commonly isolated bacteria.

	*E. coli* %	*Klebsiella pneumoniae* %	*Proteus mirabilis* %	All Isolated Bacteria %
Ampicilin	67.4	100.0	48.6	70.8
Amikacin	0.9	10.4	3.6	6.7
Ceftazidime	39.4	51.1	2.9	36
Cefixime	45.3	50.8	21.8	44.6
Ciprofloxacin	15.3	23.5	15.2	16.6
Ceftriaxon	38.0	44.3	13.1	36.4
Cefazolin	56.6	59.9	30.8	57.3
Nitrofurantoin	1.8	23.1	99.6	17.3
Gentamicin	8.7	22.7	25.2	16.6
Imipenem	5.6	16.4	85.7	16.3
Levofloxacin	13.9	6.6	14.7	12.5
Meropenem	3.8	15.5	4.0	6.3
Tobramycin	8.5	19.1	19.3	11.2
TMP/SMX	33.3	30.0	44.4	36
Piperacilin/Tazobactam	10.6	23.2	2.8	11.6
Amoxicilin/Clavulanate	44.9	44.3	11.8	43.8
Ertapenem	9.5	23.4	8.6	13.5
Cefepime	30.0	40.0	0.0	17.9
Cefuroxime	44.4	50.0	0.0	25

## Data Availability

Dataset available on request from the authors.

## Abbreviations

The following abbreviations are used in this manuscript:

UTIs	Urinary tract infections
*E. coli*	*Escherichia coli*
TMP-SMX	Trimethoprim–sulfamethoxazole

## References

[1] Mert D., Çeken S., Ertek M. (2020). İdrar yolu enfeksiyonlarında kültürden izole edilen bakteriler ve antibiyotik duyarlılıkları. Türk Hij. Deney. Biyol. Derg..

[2] Öztürk R., Murt A. (2020). Epidemiology of urological infections: A global burden. World J. Urol..

[3] Chiu C.C., Lin T.C., Wu R.X., Yang Y.S., Hsiao P.J., Lee Y., Wu T.C., Kuo S.C. (2017). Etiologies of community-onset urinary tract infections requiring hospitalization and antimicrobial susceptibilities of causative microorganisms. J. Microbiol. Immunol. Infect..

[4] Emre S., Neyzi O., Ertuğrul T. (2009). Üriner Sistem Enfeksiyonları. Pediatri.

[5] Haller M., Brandis M., Berner R. (2004). Antibiotic resistance of urinary tract pathogens and rationale for empirical intravenous therapy. Pediatr. Nephrol..

[6] Söylemezoğlu O., Hasanoğlu E., Düşünsel R., Bideci A. (2010). Üriner sistem enfeksiyonları. Temel Pediatri.

[7] Shaikh N., Morone N.E., Bost J.E., Farrell M.H. (2008). Prevalence of urinary tract infection in childhood: A meta-analysis. Pediatr. Infect. Dis. J..

[8] Berger E., Langlois V., Dipchand A., Friedman J. (2009). Nephrology. The Hospital for Sick Children Handbook of Pediatrics.

[9] Elder J.S., Kliegman R.M., Stanton B.F., St Geme J.W. (2011). Urinary tract infections. Nelson Textbook of Pediatrics.

[10] Sucu N., Aktoz-Boz G., Bayraktar Ö., Kuru F., Gültekin H. (2004). Üropatojen *Escherichia coli* suşlarının antibiyotik duyarlılıklarının yıllar içerisindeki değişimi. Klimik Derg..

[11] Yen Z.S., Davis M.A., Chen S.C., Chen W.J. (2003). A cost-effectiveness analysis of treatment strategies for acute uncomplicated pyelonephritis in women. Acad. Emerg. Med..

[12] Demir M., Kazanasmaz H. (2020). Uropathogens and antibiotic resistance in the community and hospital-induced urinary tract infected children. J. Glob. Antimicrob. Resist..

[13] Çoban B., Ülkü N., Kaplan H., Topal B., Erdoğan H., Baskın E. (2014). Çocuklarda idrar yolu enfeksiyonu etkenleri ve antibiyotik dirençlerinin beş yıllık değerlendirmesi. Turk. Pediatri Ars..

[14] Yasar A., Yaşar B., Özkan E.A., Savcı Ü. (2018). Yozgat Yöresi Çocukluk Çağı İdrar Yolu Enfeksiyonuna En Sık Sebep Olan Etkenler ve Antibiyotik Dirençleri. Bozok Tıp Derg..

[15] Konca C., Tekin M., Uckardes F., Yılmaz M., Korkmaz A. (2017). Antibacterial resistance patterns of pediatric community-acquired urinary infection: Overview. Pediatr. Int..

[16] Kömürlüoğlu A., Aykaç K., Özsürekçi Y., Can D., Aktaş N. (2018). Gram negatif idrar yolu enfeksiyonu etkenlerinin antibiyotik direnç dağılımı: Tek merkez deneyimi. J. Pediatr. Dis..

[17] Yavaşcan Ö., Sözen G., Kara D., Çetin N., Aksu N. (2005). Çocuklarda idrar yolu infeksiyonu etkenleri ve antibiyotik direnci. İzmir Tepecik Hast Derg..

[18] Elder J.S., Behrman R.E., Kliegman R.M., Jenson H.B. (2015). Urinary tract infections. Nelson Textbook of Pediatrics.

[19] Vazouras K., Velali K., Tassiou I., Dardavessis T., Maroudi M. (2020). Antibiotic treatment and antimicrobial resistance in children with urinary tract infections. J. Glob. Antimicrob. Resist..

[20] Baines G., Banjoko A., Brair A., Ghosh S., Onwudiwe N. (2020). Antibiotic resistance in urinary tract infections: A re-visit after five years and experience over two sites. Post. Reprod. Health.

[21] Al Benwan K., Jamal W. (2022). Etiology and antibiotic susceptibility patterns of urinary tract infections in children in a general hospital in Kuwait: A 5-year retrospective study. Med. Princ. Pract..

[22] Yolbas I., Tekin R., Kelekci S., Tekin A., Okur M.H., Ece A., Kadir A. (2013). Community-acquired urinary tract infections in children: Pathogens, antibiotic susceptibility, and seasonal changes. Eur. Rev. Med. Pharmacol. Sci..

[23] Çetin H., Öktem F., Örmeci A.R., Yorgancıgil B., Yaylı G. (2006). *Escherichia coli* and antibiotic resistance in childhood urinary tract infections. Suleyman Demirel Univ. Med. J..

[24] Newman T.B. (2011). The new American Academy of Pediatrics urinary tract infection guideline. Pediatrics.

[25] Roberts K.B. (2011). Urinary tract infection: Clinical practice guideline for the diagnosis and management of the initial UTI in febrile infants and children 2 to 24 months. Pediatrics.

[26] Shao H.F., Wang W.P., Zhang X.W., Li Z.D. (2003). Distribution and resistance trends of pathogens from urinary tract infections and impact on management. Zhonghua Nan Ke Xue.

[27] Tseng M.H., Lo W.T., Lin W.J., Teng C.S., Chu M.L., Wang C.C. (2008). Changing trend in antimicrobial resistance of pediatric uropathogens in Taiwan. Pediatr. Int..

[28] Edlin R.S., Shapiro D.J., Hersh A.L., Copp H.L. (2013). Antibiotic resistance patterns of outpatient pediatric urinary tract infections. J. Urol..

[29] Salduz Z., Yiğit O. (2010). Antibiotic susceptibility of bacteria isolated from children with urinary tract infections. J. Pediatr. Inf..

[30] Prais D., Straussberg R., Avitzur Y., Nussinovitch M., Harel L., Amir J. (2003). Bacterial susceptibility to oral antibiotics in community-acquired urinary tract infection. Arch. Dis. Child..

[31] Tosun S.Y., Demirel M.M., Ertan P., Aksu S. (2004). Antibiotic susceptibility of bacteria isolated from children’s urine samples. Türk. Klin. J. Pediatr..

[32] Sezgin B., Yiğit Ö., Özgürhan G., Aksoy M., Cambaz N., Beycan İ. Microorganisms responsible for urinary tract infections and antibiotic resistance in children. Proceedings of the 2nd National Pediatric Diseases Congress.

[33] Gündüz T., Tosun S., Demirel M.M., Ertan P. (2008). Antibiotic resistance in childhood urinary tract infections: Five-year results. Pamukkale Med. J..

[34] Gürgöze M.K., Doğan Y., Kizirgil A., Aşçı Toraman Z., Aygün D. (2002). Antibiotic susceptibility of bacteria isolated from children with urinary tract infections. Fırat Med. J..

[35] Cebe A., Ayvaz A., Yıldız N., Çetinkaya S. (2008). Results of urine culture in childhood urinary tract infections in Sivas: How should the initial treatment choice be made?. Van Med. J..

[36] Nassereddin Mostafavi S., Rostami S., Rezaee Nejad Y., Ataei B., Mobasherizadeh S., Cheraghi A., Shokri D. (2021). Antimicrobial resistance in hospitalized patients with community-acquired urinary tract infection in Isfahan, Iran. Arch. Iran. Med..

[37] Maraki S., Mantadakis E., Michailidis L., Samonis G. (2013). Changing antibiotic susceptibilities of community-acquired uropathogens in Greece 2005–2010. J. Microbiol. Immunol. Infect..

[38] Mantadakis E., Vouloumanou E.K., Panopoulou M., Tsouvala E., Tsalkidis A., Chatzimichael A., Gousia P. (2015). Susceptibility patterns of uropathogens identified in hospitalized children with community-acquired urinary tract infections in Thrace, Greece. J. Glob. Antimicrob. Resist..

[39] Pouladfar G., Basiratnia M., Anvarinejad M., Abbasi P., Amirmoezi F., Zare S. (2017). The antibiotic susceptibility patterns of uropathogens among children with urinary tract infection in Shiraz. Medicine.

[40] Pahissa A., Pigrau C., Sociedad Española de Ginecología y Obstetricia (2001). Tratamiento de las infecciones del tracto urinario en las embarazadas. Infecciones del Tracto Urinario en las Embarazadas.

